# Evaluation of the effect of an antenatal pelvic floor muscle exercise programme on female sexual function during pregnancy and the first 3 months following birth: study protocol for a pragmatic randomised controlled trial

**DOI:** 10.1186/s13063-019-3226-6

**Published:** 2019-02-20

**Authors:** Sahar Sadat Sobhgol, Holly Priddis, Caroline A. Smith, Hannah Grace Dahlen

**Affiliations:** 1 School of Nursing and Midwifery, Western Sydney University, Locked Bag 1797, Penrith, NSW 2751 Australia; 2 NICM Health Research Institute, Western Sydney University, Locked Bag 1797, Penrith, NSW 2751 Australia; 3 Ingham Institute, Liverpool, NSW Australia; 4 National Institute of Complementary Medicine, Campbelltown, NSW Australia

**Keywords:** Pelvic floor muscle exercise, Female sexual function, Urinary incontinence, Faecal incontinence, Childbirth, Randomised controlled trial, Protocol, Pragmatic

## Abstract

**Background:**

Sexual dysfunction can have a negative impact on women’s quality of life and relationships. There is limited information about female sexual function and treatment, particularly during pregnancy and the postpartum period. The effect of pelvic floor muscle exercise (PFME) on sexual function (SF) has not been studied adequately. The purpose of this study is to investigate the effect of antenatal PFME on female SF during pregnancy and the first 3 months following birth.

**Methods/design:**

This is a pragmatic, randomised controlled trial which will compare a structured antenatal PFME programme combined with standard antenatal care to standard antenatal care alone. Eligible women who are less than 22 weeks’ gestation will be recruited from the antenatal clinics of one hospital located in Western Sydney, Australia. A sample of 200 primiparous pregnant women who meet the inclusion criteria will be randomised to either control or intervention groups. This sample size will allow for detecting a minimum difference of 9% in the female SF score between the two groups. The duration of the PFME programme is from approximately 20 weeks’ gestation until birth. Female SF will be measured via questionnaires at < 22 weeks’ gestation, at 36 weeks’ gestation and at 3 months following birth. Baseline characteristics, such as partner relationship and mental health, will be collected using surveys and questionnaires. Data collected for secondary outcomes include the effect of PFME on childbirth outcomes, urinary and faecal incontinence symptoms and quality of life.

**Discussion:**

The findings of this study will provide more information on whether a hospital-based antenatal PFME has any effect on female SF, urinary and faecal incontinence during pregnancy and the first 3 months following birth. The study will also provide information on the effectiveness of antenatal PFME on childbirth outcomes.

**Trial registration:**

Australian New Zealand Clinical Trials registry, ACTRN12617001030369. Registered on 17 July 2017.

**Electronic supplementary material:**

The online version of this article (10.1186/s13063-019-3226-6) contains supplementary material, which is available to authorized users.

## Background

Sexual dysfunction is considered a public health concern [1, 2] and is defined as the disturbance in sexual desire and psychophysiological changes that characterise the sexual response and cause interpersonal difficulty and marked distress [3] which can affect the quality of life adversely [4]. The incidence of sexual dysfunction is estimated to range from 19 to 50% of the population, with a higher incidence in women (43%) than in men (31%) [5, 6].

It is reported in some studies that the incidence of sexual dysfunction rises to approximately 63–93% of all pregnant women [7]. Pregnancy and birth can cause significant life changes that alter previous phases of physical and emotional adjustment of couples and many women experience sexuality differently during this period [8].

About 90% of women claim to resume sexual activity 6 weeks after childbirth [9], and around 83% are reported to experience sexual problems in the first 3 months and 64% in the first 6 months following birth [9]. Acele and Karaçam [10] found that 91.3% of women experience at least one sexual problem in the postpartum period. Khajehei et al. [11] conclude that 64.3% of Australian women reported sexual dysfunction in the first year after birth and 70.5% expressed sexual dissatisfaction.

Sexual activity involves a complicated interaction of physiological, psychosocial and behavioural components in and between individuals [12]. Among several factors associated with sexual dysfunction (such as sleep deprivation, breastfeeding, fatigue, etc.) [9], loss of pelvic muscle strength is one of the physical changes which occurs in the postpartum period and may be present even years afterwards. This can result in pelvic pain, urinary incontinence, cystocele, rectocele and lack of sexual satisfaction [13]. Having healthy pelvic floor muscle strength (PFMS) is important for satisfactory genital arousal and orgasm. Weak muscles may not provide adequate stimulation and arousal, thus hindering orgasmic potential [14].

Women seeking assistance with sexual dysfunction find few options of care available to them [15]. Treatment of female sexual dysfunction is still an unmet clinical need. There are no Food and Drug Administration-approved treatments available for women [16]. PFME has been proposed to be a potential factor impacting on female SF. There may be a psychosocial effect of PFME, such as improved self-acceptance, body awareness and satisfaction [17, 18]. PFME contributes to increased blood flow to the pelvis and enhanced vaginal and clitoral receptivity and responsiveness, providing pleasure during intercourse for both partners. Some studies have shown that strong pelvic floor muscles may be associated with better orgasmic and arousal potentials, desire, excitement and vaginal lubrication [3, 8]. Despite this theoretical background there are limited randomised controlled trials (RCTs) on the effect of PFME on female sexual dysfunction [3], particularly during pregnancy and the postpartum period. Pauls et al. [8] suggested that pelvic floor muscle (PFM) training should be the first-line treatment for stress urinary incontinence and pelvic organ prolapse. However, there is a need for RCTs to test whether PFME can reduce sexual dysfunction [8].

The pelvic floor is a rich and dynamic system of interconnected muscles, nerves and ligaments that play a major role in maintaining urinary continence in childbearing women. During birth, the baby’s head stretches the surrounding structures and this stretching may result in damage to the muscles, nerves and connective tissues of the pelvic floor. An association between childbirth and urinary incontinence (UI) may be exacerbated by the use of forceps and can lead to long-term pelvic floor muscle dysfunction and urinary or faecal incontinence [19–21]. PFME before the labour and birth could produce strong and well-controlled muscles, reducing incontinence and even potentially reducing fatal malposition and malpresentation [22, 23]. Studies that examined the effect of PFME on labour show mixed results. There are studies demonstrating that women randomised to antenatal PFME had a lower rate of prolonged second stage of labour, less breech presentations and a lower episiotomy rate. The authors concluded that PFME appeared to facilitate rather than obstruct labour [23]. However, another study found no effect of PFME on the duration of the second stage of labour or the rate of instrumental birth [24]. There is additional evidence that PFME may have an effect on reducing faecal incontinence particularly at 1 year postpartum [25].

Therefore, a pragmatic parallel, randomised controlled trial was designed to investigate the effect of antenatal PFME on SF during pregnancy and the first 3 months following birth. The secondary objectives of this study are to investigate the effect of PFME on childbirth outcomes, urinary and faecal incontinence symptoms and specific quality-of-life measures during pregnancy and the first 3 months following birth.

## Methods

### Study design

A pragmatic, parallel RCT will be conducted to compare the effect of a structured antenatal PFME programme combined with routine antenatal care to routine standard antenatal care alone. The trial design incorporates treatment flexibility with the framework of a self-designed (semi-standardised) structured protocol of PFME. Any changes that need to be made to the trial protocol will be communicated to all investigators, the ethics committees and the trial registry. The protocol (version 1) has been designed in accordance with Standard Protocol Items: Recommendations for Interventional Trials (SPIRIT) guidelines for interventional trials [26] (Additional file 1) and will be conducted in accordance with the Declaration of Helsinki (1964) and the International Conference on Harmonization Good Clinical Practice (1996).

### Eligibility criteria

Low-risk primiparous women who present to the antenatal clinic for antenatal care will be approached to participate in the study. Women will be eligible if they are primiparous, over 18 years old, ≤ 22 weeks’ gestation, have a singleton pregnancy, anticipating a vaginal birth and have no history of urinary incontinence, pelvic surgery or pelvic organ prolapse at baseline. They also should have no previous history of depression, mental illness, alcohol and drug use or domestic violence and they need to be able to read, understand and communicate in English. Participants in the treatment group must also agree not to perform a PFME programme other than that provided in this study. Women will be excluded if they are over 22 weeks’ gestation, planning to give birth via caesarean section at the time of booking, multiparous, have a multiple pregnancy, have complicated pregnancies (type 1 and type 2 diabetic, vaginal bleeding, baby with abnormalities, etc.) and have known pelvic floor muscle dysfunction. Women who are not able to read and understand English to answer the questionnaires will also be excluded from this study.

### Recruitment, setting and informed consent

The recruitment will be undertaken at the antenatal clinics of a tertiary, teaching public hospital located in Western Sydney, Australia. The region includes a diverse population from diverse ethnic, cultural and economic backgrounds. Women will be recruited at the time of booking into the hospital or at their second antenatal visits when they are less than 22 weeks’ gestation. Pregnant women will be introduced into the study via flyers available in the antenatal clinics. The researcher will attach flyers to the files of all primiparous women less than 22 weeks’ gestation who are attending their first or second antenatal appointment. Midwives booking the women into the antenatal clinics will refer the women to the researcher, who will then provide women with detailed information about the research and confirm they are interested in joining the study. All women will be screened again to see whether they are eligible, and they will be provided with a participant information sheet and asked to provide written consent.

### Randomisation and blinding

It is not possible for study participants, and the first researcher (SSS) delivering the intervention, to be blind to the group allocations in this study. The other care providers, including midwives and doctors, will be blind to the group allocations. The first researcher (SSS) will assign women to the intervention or control groups using a centralised, remote computer-generated randomiser called Sealed Envelope [27]. To minimise the risk of detection bias, every woman will be issued with a unique four-digit study code. Therefore, the researcher (SSS) will be blinded to the participants’ responses using the study code during data entry. Additionally, data will be collected electronically and submitted by the women directly into the Red Cap database, which will further decrease the risk of bias. The first researcher (SSS) will be kept blinded to the group allocations during data analysis and the statistician will conduct the analysis with the researcher. There will be a treatment and control ratio of 1:1. All data including electronic and hardcopies will be destroyed 5 years after completion of the study according to NSW health policy.

### Treatment and assessment schedule (participant’s timeline)

**Fig. 1:**
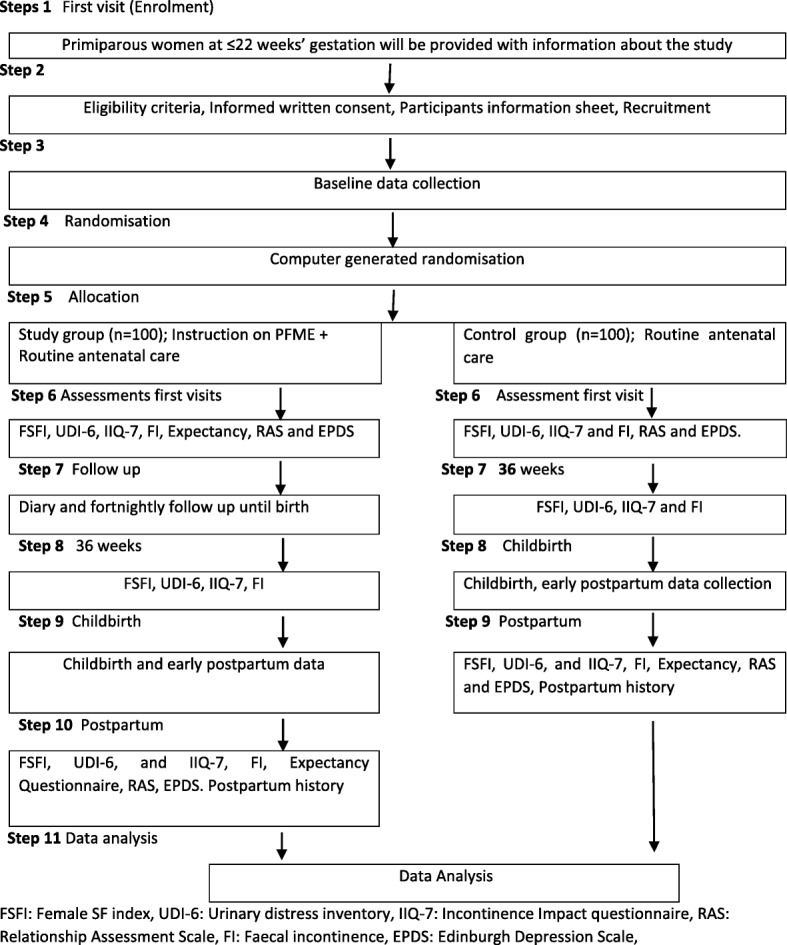
Pragmatic randomised controlled trial flowchart

The study period will run from approximately 20 weeks’ gestation until 3 months following birth. The duration of the PFME programme is from approximately 20 weeks’ gestation until birth. to assess the comparibility of the study groups, baseline information about demegraphic data, medical, surgical, psychological and family histories will be obtained from the hospital databse. Figure 1 outlines the process of the study.

### Intervention

#### Background

Various PFME protocols in non-pregnant women have been used in the literature. However, the number of repetitions and the duration of contractions and rest between series vary. The recommended frequency of PFME ranges from two to three times a week for up to a 3-month period, an amount of time necessary to obtain minimum hypertrophy and consequently muscle strength. Although various studies have confirmed the efficacy of PFME in the treatment of urinary incontinence, reports regarding PFME during pregnancy and the postnatal period are scarce [28]. According to Bø [29], those women who performed PFME with supervised training gain the most beneficial effect compared with those who do not receive training or supervision. About 200 million women around the world are not familiar with PFME. Moreover, it is reported that approximately 50% of women do not perform this exercise correctly [30].

PFME has been recommended as a first-line treatment for pelvic floor dysfunction, including urinary incontinence [14]. However, to date, there is no consensus about the most effective PFME regime to recommend to women, even from the most rigorously designed studies on urinary incontinence [31]. There is also conflicting information about whether using biofeedback to teach PFME is more effective than PFME alone. The study by Finnbogadóttir et al. [32] showed that women with a poor ability to contract their PFM had better PFMS when they were trained using biofeedback methods compared with those who received PFME alone. In contrast, Harvey [33] reported that antenatal PFMEs with biofeedback taught by trained personnel do not significantly reduce the incidence of postpartum urinary incontinence or improve PFMS in the short term. Fitz et al. [34] found that PFME alone led to a superior but not significant difference in the function of PFM when compared to PFME with biofeedback. This suggests that PFME with biofeedback is no more effective than PFME alone [34]. Compliance rates with PFME continue to be an issue [35, 36]. To increase the effectiveness of PFME, the recommended strategies include; appropriate follow up, feasibility of the program, training program, and patient-related actors such as motivation and commitment [35, 36].

#### Intervention used in this study

**Fig. 2:**
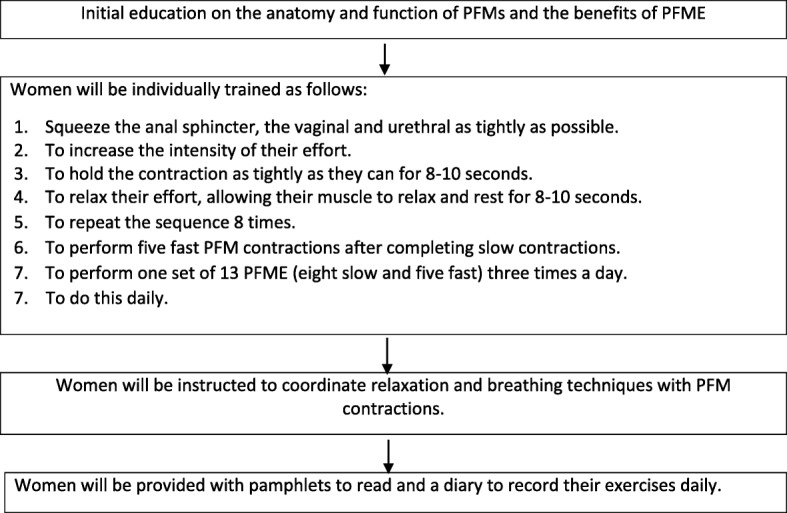
Instructions for women. PFM pelvic floor muscle, PFME pelvic floor muscle exercise

In this study, all women will receive standard antenatal care. In addition to routine antenatal education and care, the women in the intervention group will receive an initial education session on the function of PFMs and the benefits of PFME as well as how to perform PFME. Women will be provided with an additional pamphlet to read (version 06; published by the Continence Foundation of Australia) [37]. The duration of treatment is approximately from 20 weeks’ gestation until birth. The first training session will be conducted by the researcher and is designed to take 20–30 min. To design the PFME programme in this study, the researchers used a combination of information obtained from the physiotherapy department in the intended hospital setting as well as the recent literature available on PFME programmes. The final PFME programme in this study was developed using six references: effective functional motor activation patterns (the Knack method) [38, 39] and the studies carried out by Elbegway et al. [13], Mørkved et al. [22], Mørkved and Bø [40] and Alves et al. [41] (Fig. 2).

The women will be instructed how to lift their PFM up and inwards around the urethra, vagina and rectum, squeeze as hard as they can and hold it for 8–10 s before relaxing the muscles gently. Women will be asked to keep breathing in and out during the contractions [40]. Women will be instructed to perform PFME in different positions so they can choose the most comfortable position to practise PFME at home (Table 1). Performing the proposed PFME at home is estimated to take 15 min.

- Sit with your legs apart and your back straight. Lift upwards and inwards around the openings in the pelvic floor [40].
- Stand with your legs apart, and check that the buttock muscles are relaxed while you squeeze the PFMs [40].

**Table 1:** Pelvic floor muscle exercise sequences

Gestational stage	Position adopted	Type of muscular contraction	Number of contractions	Contraction sustaining time (s)	Muscle relaxation time after contraction (s)
From 20 weeks until birth	Standing	Slow	8–10	8–10	8–10
		Fast	5	1	–
	Sitting	Slow	8–10	8–10	8–10
		Fast	5	1	–
	Lying down	Slow	8–10	8–10	8–10
		Fast	5	1	–

Women will be instructed on the Knack method (Table 2). They will be taught to contract their PFMs before coughing, sneezing, lifting or bending. Women will also be asked to stop the flow of urine when they are urinating. If it is difficult to stop the initial flow, they can test themselves towards the end of urination, which is much easier. This is only a test to see whether they are using the muscles correctly. They are asked not to use urination for training, as this can interfere with the ability to empty their bladder completely [35, 39].

**Table 2:** Knack method

Aim	3 sets of 13 pelvic floor muscle contractions lasting 8–10 s daily
Triggers (red stick-up dots)	Shower, teeth, meals, queues and so forth
The Knack	Functional bracing: grip up before cough, sneeze, lift, bend
Postnatally	As above and also incorporating good bladder habits

In order to assess the compliance rate, women in the intervention group will be provided with a diary to record their exercises daily. Women will be followed up fortnightly via text messages as reminders. Red stick-up spots will be used to remind women to do the exercises as this has been demonstrated to be effective [38].

Both study and control groups will receive routine antenatal care during pregnancy. The routine antenatal care includes verbal advice on PFME given by midwives to women at 28 weeks’ gestation. The advice is given in one antenatal visit and is known to be inconsistent and brief, as there is no structured PFME programme available. The available antenatal classes also do not cover a structured antenatal PFME training with follow up. The physiotherapy service is available to the women who are referred to a physiotherapist to receive specific physical therapy treatment.

In this study, women in the control group will not receive training on PFME from the researcher; however, they will not be discouraged from performing PFMEs. There is no dietary or lifestyle requirement or restriction as a result of participating in this study. Women in the intervention group are required to follow the PFME protocol provided by the researcher and to avoid using a different PFME protocol.

If women develop any complications related to pregnancy, they will be allowed to withdraw from the study if they are advised to do so by a doctor.

### Outcome measures and data collection

**Fig. 3:**
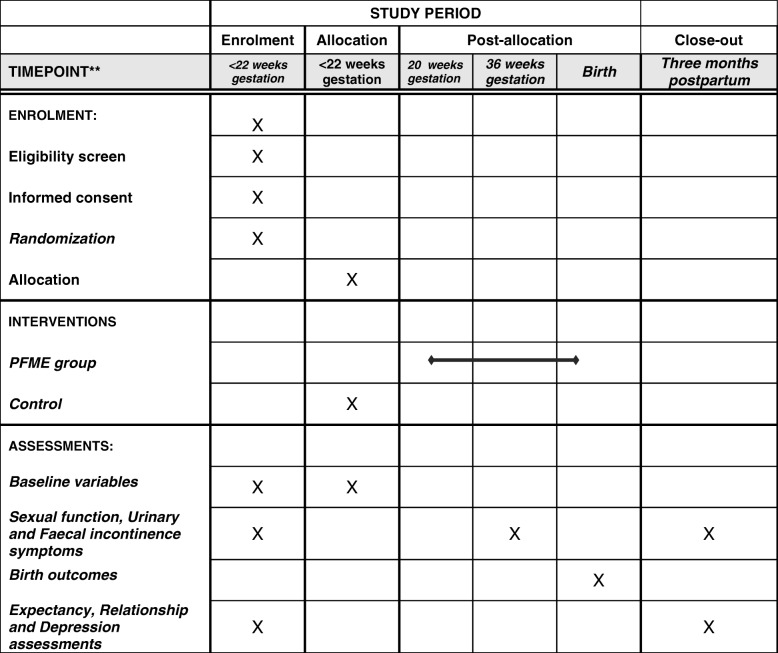
The schedual of enrollment, interventions and assessment

The list of data collection instruments and time of data collection are presented in Table 3 and Fig. 3. The primary outcome of this study is the measurement of female SF during pregnancy and at 3 months postpartum which will be assessed using the Female Sexual Function Index (FSFI) [42]. The FSFI is a valid and reliable [42] multidimensional self-reported instrument for the assessment of female SF. It consists of 19 questions grouped into six domains: desire, arousal, lubrication, orgasm, satisfaction and pain. A value of 0–5 is attributed to each answer. The scores range from 2 to 36 and the lower the score, the worse the SF [42]. There are cut-off points to predict female sexual dysfunction. In 2006, Weig et al. [1] validated the FSFI in the USA and found that a cut-off score of 26.55 was enough to classify 70% of women with sexual dysfunction in the normal population. This score cannot be used for specific groups such as pregnant women, postpartum women, menopausal women and so forth [1]. SF will be assessed at 20 and 36 weeks’ gestation and at 3 months following birth.

**Table 3:** Data collection instruments and time of data collection

	Collection time							
	Booking		36 weeks’ gestation		Birth		3 months after birth	
Data and tools	CG	IG	CG	IG	CG	IG	CG	IG
Primary outcomes								
SF (FSFI)	√	√	√	√	–	–	√	√
Secondary outcomes								
Urinary incontinence (UDI-6)	√	√	√	√	–	–	√	√
Faecal incontinence (FI questionnaire)	√	√	√	√	–	–	√	√
Specific quality of life (IIQ-7)	√	√	√	√	–	–	√	√
Childbirth outcomes (Obstetrix Database)	–	–	–	–	√	√	–	–
Confounding variables								
Depression (EPDS)	√	√	–	–	–	–	√	√
Relationship (RAS)	√	√	–	–	–	–	√	√
Expectancy questionnaire	√	√	–	–	–	–	–	–
Baseline data (Obstetrix Database)	√	√	–	–	–	–	–	–
Antenatal history (Obstetrix Database and survey)	√	√	√	√	–	–	–	–
Postpartum history (Obstetrix Database and survey)	–	–	–	–	–	–	√	√

*CG* control group, *IG* intervention group, *SF* sexual function, *FSFI* Female Sexual Function Index, *UDI-6* Urinary Distress Inventory, *FI* Waxner short form of faecal incontinence, *IIQ-7* Incontinence Impact Questionnaire, *EPDS* Edinburgh Depression Scale, *RAS* Relationship Assessment Scale

The secondary outcome endpoints will be examined as follows:

1. Childbirth outcomes will include duration of the first, second and third stages of labour, fetal positions and presentation at birth; mode of birth; episiotomy and perineal trauma (1st, 2nd, 3rd and 4th degree perineal lacerations); and fetal outcomes including the APGAR score, head circumference, length and weight. Data regarding childbirth outcomes and fetal outcomes will be obtained from the hospital database after birth.
2. Urinary incontinence symptoms and specific quality-of-life measures will be assessed using the short forms of the Urogenital Distress Inventory (UDI-6) [43] and the Incontinence Impact Questionnaire (IIQ-7) [43] at the time of booking at 20 weeks’ gestation, at 36 weeks’ gestation and also at 3 months following birth. The short forms of the UDI-6 and IIQ-7 are composed of six and seven questions, respectively. They are validated self-report questionnaires and have been shown to have a high degree of correlation with the longer forms of these questionnaires. Both questionnaires are recommended by the second international consultation on incontinence [44]. The UDI-6 is a six-item symptom inventory, specific to symptoms associated with lower urinary tract dysfunction, and combines information on stress, obstructive and discomfort symptoms. The IIQ-7 is a seven-item life-impact assessment instrument specific to UI, and covers separate domains of physical activity, travel, social/relationships and emotional health. Both measures were developed for self-administration and are intended to be used in combination. Women rate how much they experience impaired function of UI and the extent to which this UI affects their daily functioning with four response options per item (0 = “not at all”; 1 = “slightly”; 2 = “moderately”; 3 = “greatly”). For this study, we maintained the scoring procedure as described by the original developer: if more than two items are missing, a total score is not to be calculated. The mean score of items is multiplied by $$ 33\raisebox{1ex}{$1$}\!\left/ \!\raisebox{-1ex}{$3$}\right. $$ to convert to a 0–100 scale. Higher scores indicate more symptom distress (UDI-6) or more impact on daily life (IIQ-7) [45].
3. Faecal incontinence (FI) symptoms will be assessed using a Waxner short form of faecal incontinence questionnaire [46] and has been validated in Australia [47]. The Waxner classification is a 20-point scale used to determine the severity of faecal incontinence and is widely used in Europe [46]. The Waxner questionnaire consists of five questions addressing solid, liquid, gas, changing underwear and lifestyle. Excellent or a score of 0 means continent to liquid and solid stool. Fair means incontinent to liquid and flatus. Poor or a score of 20 means total incontinence [46].

In order to examine the baseline characteristics that might interfere with the effect of PFME on SF, women in both control and intervention groups will be asked about depression symptoms (using the Edinburgh Postnatal Depression Scale (EPDS) questionnaire) [48]. The 10-question EPDS is a valuable and efficient way of identifying patients at risk for “perinatal” depression. Scores above 12 require further assessment and appropriate management as the likelihood of depression is high. Any woman who scores 1, 2 or 3 on item 10 requires further evaluation before leaving the office to ensure her own safety and that of her baby [48].

The other factor that can interfere with the effect of PFME on SF is the relationship with the partner. In this study, a relationship questionnaire [49] will be used at the time of enrolment and at 3 months following birth. The Relationship Assessment Scale (RAS) used in this study consists of seven questions. Scores could range from 1 (low satisfaction) to 5 (high satisfaction). The scores are added up and divided by 7 to get a mean score. The RAS is internally consistent and is solidly and consistently related to measures of relevant constructs such as love and self-esteem [49].

Women in the intervention group will also be provided with the expectancy questionnaire [50] at the time of enrolment. As there is no standard expectancy questionnaire available, for the purpose of this study a questionnaire was designed based on a questionnaire extracted from a study by Devilly and Borkovec [50]. This questionnaire will provide information on women’s expectation of treatment. Belief usually has two aspects to it: (1) what one thinks will happen; and (2) what one feels will happen. The original questionnaire has demonstrated high internal consistency within each factor and good test–retest reliability [50].

Data to be collected from the woman at baseline include: age, gestational age, education, economic status, country of birth, relationship status, smoking habits, remedies or medication history, medical, surgical and psychological history (such as mental illness or depression), family history including domestic violence and alcohol and drug abuse. Antenatal, birth and postpartum history will be obtained from the hospital database and a self-designed survey. The survey will be completed at the time that questionnaires are given to women at the time of booking, at 36 weeks’ gestation, and at 3 months after birth.

To assess the compliance rate, women will be given a diary to record their PFME daily. These data will be collected and recorded monthly by the researcher.

All questionnaires are self-report questionnaires and will be delivered electronically through the REDCap electronic system [51] to women unless they choose to receive hardcopies. The first researcher (SSS) will collect the data. All missing data will be reported with reasons given and patterns of occurrence explored. If women withdraw from the study, no more additional information will be collected; however, the personal information already collected will be retained to ensure that the results of the research project can be measured properly and to comply with the law.

### Safety monitoring

If women raise any concerns about their health during recruitment and throughout the research process, they will be provided with numbers for counselling by the research team and they will be provided with appropriate referral to health care professionals to receive additional treatment and care.

PFME is currently part of antenatal and postnatal education and is considered a safe practice. There are no adverse effects previously reported in the literature; however, any adverse events arising during the study will be documented and reported to the Human Ethics Research Committee.

### Sample size

The sample size of this study was calculated based on the recent research available on the impact of PFME on SF in the postpartum period. Using information from this study [52], a total FSFI score of 28.9 ± 4.54 in the intervention group after PFME and 26.6 ± 4.40 in the control group and a standard deviation of 4.54 in both groups, a sample size of minimum of 62 in each arm would be required to detect a 9% difference in FSFI scores between groups (80% power and *α* = 0.50). The treatment and control ratio is 1. Allowing for 20% attrition during pregnancy, around 160 women are needed to be recruited. However, considering the possibility of a higher rate of withdrawal at 3 months after birth and anticipating 30% attrition after birth, 200 women will be randomised.

### Data management

Data will be collected via an electronic data management system (REDCap) and will be stored securely on a password-protected computer in the hospital setting. Data will be linked to a study code and de-identified data will be used for data analysis. All of the hardcopies will be de-identified using a study code and will be stored securely in the hospital setting. All data will be destroyed 5 years after completion of the study.

### Statistical analysis

Analysis of the primary and secondary outcomes will be based on an intention-to-treat basis, which will include withdrawal and losses to follow up. Descriptive statistics including numbers and proportions, and means and standard deviations will be used. Primary analysis including chi-square and Student’s *t* tests will be used to examine group differences in the primary and secondary outcomes. Repeated ANOVA will be used in order to assess within-patient correlation. Effect sizes will be reported with 95% confidence intervals, and results will be considered significant if *P* < 0.05. Data will be analysed using SPSS.

## Discussion

To date, there is an international consensus that pelvic floor muscle training (PFME) should be the first-line treatment for stress urinary incontinence (SUI) and pelvic organ prolapse (POP). There is, however, no consensus on either prevention or treatment of symptoms related to female sexual dysfunction [3, 18]. Much of the published research in this area addresses the efficacy of treatment protocols, which aim to cure or improve urinary incontinence once it has manifested. Therefore, this study provides the opportunity to assess the preventive effect of antenatal PFME on urinary and faecal incontinence during pregnancy and in the postpartum period. This study will test the effectiveness of a hospital-based PFME programme alone without biofeedback with emphasis on the continuity of care and motivation, without using vaginal examination or invasive methods during pregnancy. It also will provide more information on the effect of antenatal PFME on childbirth outcomes. Findings from this study will help to establish an evidence base for the use and effectiveness of hospital-based antenatal PFME to improve primiparous women’s quality of life, SF, childbirth outcomes, and urinary and faecal incontinence symptoms.

The limitation of this study is that the PFMS will not be assessed both before and after intervention, but if this PFME programme is shown to be effective then it will be even more feasible to use in clinical practice. There is no biofeedback method being used in this study and there will be no digital examination to teach or assess whether women are contracting their PFM correctly. The fortnightly follow up on PFME will cease at birth and does not continue after birth. The women will be followed up only until 3 months postpartum and the follow up will not continue beyond that period. The participants are all primiparous so the effect of antenatal PFME on multiparous pregnant women will not be assessed in this study.

## Trial status

Recruitmnet commenced on 11 Februray 2018. It is expected to be completed by 31 September 2018. Data colletcion will be completed by 31 April 2019.

## Additional file

Additional file 1: SPIRIT 2013 checklist: recommended items to address in a clinical trial protocol and related documents. (DOC 116 kb)

## Abbreviations

PFM: Pelvic floor muscle
PFME: Pelvic floor muscle exercise
PFMS: Pelvic floor muscle strength
RCT: Randomised controlled trial
SF: Sexual function
